# The Complex Biogeography of the Plant Pathogen *Xylella fastidiosa*: Genetic Evidence of Introductions and Subspecific Introgression in Central America

**DOI:** 10.1371/journal.pone.0112463

**Published:** 2014-11-07

**Authors:** Leonard Nunney, Beatriz Ortiz, Stephanie A. Russell, Rebeca Ruiz Sánchez, Richard Stouthamer

**Affiliations:** 1 Department of Biology, University of California Riverside, Riverside, California, United States of America; 2 Centro de Investigacion en Biologıa Celular y Molecular, Universidad de Costa Rica, San José, Costa Rica; 3 Department of Entomology, University of California Riverside, Riverside, California, United States of America; Oklahoma State University, United States of America

## Abstract

The bacterium *Xylella fastidiosa* is a plant pathogen with a history of economically damaging introductions of subspecies to regions where its other subspecies are native. Genetic evidence is presented demonstrating the introduction of two new taxa into Central America and their introgression into the native subspecies, *X. fastidiosa* subsp. *fastidiosa*. The data are from 10 genetic outliers detected by multilocus sequence typing (MLST) of isolates from Costa Rica. Six (five from oleander, one from coffee) defined a new sequence type (ST53) that carried alleles at six of the eight loci sequenced (five of the seven MLST loci) diagnostic of the South American subspecies *Xylella fastidiosa* subsp. *pauca* which causes two economically damaging plant diseases, citrus variegated chlorosis and coffee leaf scorch. The two remaining loci of ST53 carried alleles from what appears to be a new South American form of *X. fastidiosa*. Four isolates, classified as *X. fastidiosa* subsp. *fastidiosa*, showed a low level of introgression of non-native DNA. One grapevine isolate showed introgression of an allele from *X. fastidiosa* subsp. *pauca* while the other three (from citrus and coffee) showed introgression of an allele with similar ancestry to the alleles of unknown origin in ST53. The presence of *X. fastidiosa* subsp. *pauca* in Central America is troubling given its disease potential, and establishes another route for the introduction of this economically damaging subspecies into the US or elsewhere, a threat potentially compounded by the presence of a previously unknown form of *X. fastidiosa*.

## Introduction

*Xylella fastidiosa* is a pathogenic bacterium native to the Americas that infects the xylem of many species of plant host. It is transmitted among plant hosts by xylem feeding insects, typically leafhoppers [1]. *X. fastidiosa* is divided into five subspecies each of which has a characteristic and largely non-overlapping host range [2]–[4]. Among these plant hosts are several economically important plants, most notably grapevine, whose infection by *X. f.* subsp. *fastidiosa* in North and Central America causes Pierce's disease, and citrus and coffee, whose infection by *X. f.* subsp. *pauca* in parts of South America (primarily Brazil and Argentina) causes citrus variegated chlorosis (CVC) and coffee leaf scorch, respectively.

Genetic data indicate that four of the five subspecies have allopatric origins, with *X. f.* subsp. *multiplex* native to North America (based on data from the US), *X. f.* subsp. *fastidiosa* native to Central America (based on data from Costa Rica), and *X. f.* subsp. *pauca* native to South America (based on data from Brazil), with the origins of *X. f.* subsp. *sandyi*, which appeared as a single genotype in the US in the 1980s, remaining uncertain [10], [11], [13]. Approximate divergence times suggest that *X. f.* subsp. *pauca* diverged from the other forms more than 50,000 years ago, followed by *X. f.* subsp. *multiplex*, and then *X. f.* subsp. *fastidiosa* and *X. f.* subsp. *sandyi* around 20,000–40,000 years ago [3], [10].

In the recent past, this geographical pattern has become less clear due to the movement of subspecies, presumably due to human-mediated introductions [10], [11], [13]. These introductions have resulted in varying levels of inter-subspecific genetic introgression [10]–[12], including the massive mixing of the *X. f.* subsp. *multiplex* and *X. f.* subsp. *fastidiosa* genomes within the US that led to a distinct taxon, provisionally named *X. f.* subsp. *morus* [4].

Anthropogenic movement of subspecies has been a primary factor in the epidemiology of *X. fastidiosa* caused diseases. Nunney et al. [11] established that a single strain of *X. f.* subsp. *fastidiosa* introduced into the US from Central America is ancestral to all of the Pierce's disease causing bacteria now found within the US. A second introduction of *X. f.* subsp. *fastidiosa* into the US, although ultimately unsuccessful, has been implicated in a large-scale intersubspecific homologous recombination (IHR) event with the native *X. f.* subsp. *multiplex* leading to both *X. f.* subsp. *morus* [4], the newly proposed subspecies that is the only form known to naturally infect mulberry, and the recombinant group of *X. f.* subsp. *multiplex*, a group that infects a range of hosts but notably includes the only strains known to infect blueberry [12], [13].

In addition, it has been proposed that IHR created the genetic variation necessary for the invasion of citrus and coffee [10] by *X. f.* subsp. *multiplex* in Brazil and Argentina. CVC was first reported in 1987 [5] and has resulted in major economic losses in Brazil [6], [7], while coffee leaf scorch was first documented in 1995 [8]. The coffee and citrus isolates do not typically cross infect [9] and show genetic differences [9], [10]; however, both show evidence of substantial genetic introgression from another subspecies, tentatively assumed to be the North American subspecies, *X. f.* subsp. *multiplex* [10]. The hypothesis that *X. f.* subsp. *pauca* became a serious pathogen of citrus following this introgression accounts for the long CVC-free history of citrus in South America and predicts that the “pure” *X. f.* subsp. *pauca* does not cause serious infection in citrus or coffee. However, this proposal remains to be tested since no isolates of *X. f.* subsp. *pauc*a that lack the introgressed material have been found, perhaps because its native hosts are still unknown.

Thus, there is evidence of the movement of *X. fastidiosa* subspecies from Central America to North America, and from North America to South America, and strong circumstantial evidence exists that this movement has led to the invasion of new hosts. These conclusions were based upon data derived from the simple but powerful technique of multilocus sequence typing (MLST). In all cases, the analysis using additional sequence data [10], [13] or whole genome data [11] has fully validated the conclusions derived from MLST.

Here MLST data are presented demonstrating another example of subspecific mixing, this time in Central America. Specifically, the hypothesis that South American *X. f.* subsp. *pauca* had been introduced into Central America, a region where *X. f.* subsp. *fastidiosa* is native [11], is explored. The genetic analysis not only supports this hypothesis, it also provides evidence for the introgression of genetic material from an undescribed form of *X. fastidiosa* that may also have been introduced from South America into Central America.

## Materials and Methods

### MLST analysis

The analysis was based 38 isolates from Costa Rica obtained from symptomatic plants as described in Montero-Astua et al [16]. Details of 24 of these isolates are provided elsewhere [11], [16]; the remainder were isolated by BO and others at the University of Costa Rica. DNA was extracted from cultures derived from these isolates by us at the University of Costa Rica and shipped to the University of California Riverside where we typed the isolates using the MLST scheme developed for this species [14], [15], plus we sequenced the non-MLST *pilU* locus. The seven MLST genes (parts of housekeeping genes *leuA*, *petC*, *malF*, *cysG*, *holC*, *nuoL*, and *gltT*) and the cell surface protein coding gene (*pilU*) were sequenced using the methods described in Yuan et al. [15]. MLST for 24 of these isolates has been previously published [11], Using the MLST protocol, any novel allele identified at one of the MLST loci was identified by the next available number defining alleles at that locus, expanding the database of known *X. fastidiosa* variation (maintained at www.pubmlst.org/xfastidiosa). Consequently, each isolate was described by its allelic profile, consisting of a set of seven numbers defining the alleles at each of the seven loci. Each unique allelic profile was assigned a sequence type (ST) number.

The sequence data were used to identify any genetically atypical isolates. An atypical isolate is defined as one that carried one or more alleles that did not cluster genetically with alleles from *X. fastidiosa* subsp. *fastidiosa* and instead clustered more closely with another subspecies.

### Analysis of genetic distance

Because there is extensive evidence of recombination in *X. fastidiosa*, the genetic differences among the STs and gene sequences either alone or in combination with other genes are best documented by genetic distance rather than phylogenetic models, which assume clonality. The distance trees with relevant bootstrap values (from 1000 replicates) were constructed using the programs Seqboot, Dnadist, Fitch, and Consense from Phylip 3.69 [17]. In the analysis, the two known indels (6 bp and 30 bp) in *nuoL* were given weights equivalent to one and three transversions, respectively. All trees were rooted using sequence data from the Taiwanese pear leaf scorch strain [18].

### Nucleotide sequence accession numbers

The MLST data are available at the MLST website (http://pubmlst.org/xfastidiosa). The gene sequences for the MLST alleles analyzed in the present study are available at GenBank under the following accession numbers: for *leuA*, allele: 7 =  FJ610159; 11 =  HM243595; 13 =  HM243596; for *petC*, allele: 1 =  FJ610165; 6 =  FJ610168; 9 =  HM243598; for *malF*, allele: 10 =  FJ610180; 11 =  FJ610180; 16 =  KM077165; for *cysG*, allele: 15 =  HM243603; 23 =  HM243605; 24 =  KM077166; for *holC*, allele: 10 =  FJ610197; 16 =  KM077167; 20 =  HM596024; for *nuoL*, allele: 5 =  HM243611; 12 =  HM243613; 16 =  KM077168; for *gltT*, *allele*: 1 =  FJ610213; 10 =  FJ610219; 14 =  KM077169; and for *pilU*, allele: 11 =  FJ610230; 12 =  FJ610229; 18 =  KM188062; 26 =  KM077170; 27 =  KM077171; 28 =  KM077172.

## Results

Previous genetic typing of 24 isolates from Costa Rica by MLST classified them all as *X. f.* subsp. *fastidiosa* [11]. Subsequent typing of 14 additional Costa Rica isolates revealed nine with atypical alleles at one or more of the eight loci we routinely sequence (seven used for MLST plus *pilU*). These isolates with atypical alleles defined two new STs (six ST53, three ST61) and, in addition, one of the original isolates (PD0411) that had been typed as ST47 [11], was found to carry an atypical *pilU* sequence (Table 1). Note that the high frequency of atypical isolates (10/38) is largely due to repeated sampling of oleander (6 isolates) to determine if additional atypical genotypes could be found.

**Table 1:** Genetic typing of isolates of *X. fastidiosa* from Costa Rica indicating, in addition to the genetic data, the plant host sampled and the location.

Isolate name (host)	ST^a^			MLST loci^b^					non-MLST locus	alias	Location (year)
		*leuA*	*petC*	*malF*	*cysG*	*holC*	*nuoL*	*gltT*	*pilU*		
CIT0208 (citrus)	*61*	11	9	11	15	16-unk	12	10	11	C.sp. Ic1	Santa Elena, San José Province (2007)
COF0395 (coffee)	*61*	11	9	11	15	16-unk	12	10	11	SD11	Santo Domingo, Heredia Province (2009)
COF0413 (coffee)	*61*	11	9	11	15	16-unk	12	10	11	SD18 coffee	Santo Domingo, Heredia Province (2009)
COF0407 (coffee)	*53*	7-p	6-p	16-p	24-unk	10-p	16-unk	14-p	27-p	C18	Curridabat, San José Province (2009)
OLS0409 (oleander)	*53*	7-p	6-p	16-p	24-unk	10-p	16-unk	14-p	27-p	Oleander1	San José, San José Province (2009)
OLS0473 (oleander)	*53*	7-p	6-p	16-p	24-unk	10-p	16-unk	14-p	27-p	CSP5	San Pedro, San José Province (2010)
OLS0478 (oleander)	*53*	7-p	6-p	16-p	24-unk	10-p	16-unk	14-p	27-p	Narciso 1	Sabanilla, San José Province (2010)
OLS0479 (oleander)	*53*	7-p	6-p	16-p	24-unk	10-p	16-unk	14-p	27-p	Narciso 3	Sabanilla, San José Province (2010)
OLS0480 (oleander)	*53*	7-p	6-p	16-p	24-unk	10-p	16-unk	14-p	27-p	Oleander3	Sabanilla, San José Province (2010)
PD0411^c^ (grape)	*47*	13	1	10	23	20	5	1	28-p	5271 grape	La Uruca, San José Province (2009)

a ST: Sequence Type, defined by each unique combination of alleles at the MLST loci.

b Each allele at a locus is given a number. Alleles are considered to be from *X. f.* subsp. *fastidiosa* unless they are identified as -p (*X. f*. subsp. *pauca*) or -unk (unknown origin).

c MLST typing from Nunney et al [11].

**Figure 1:**
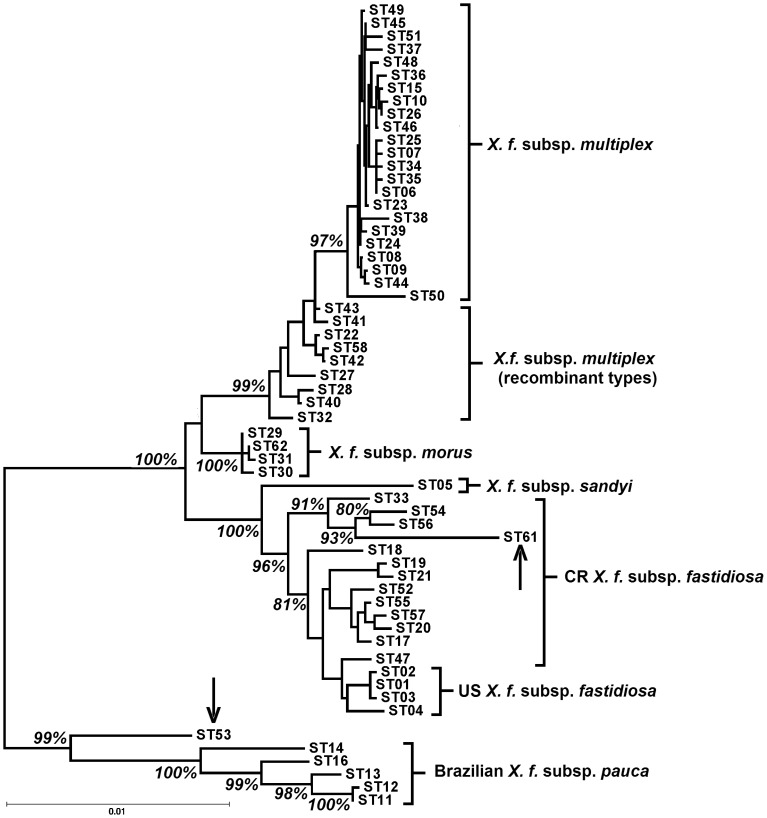
Distance tree showing the relationship of the two atypical Sequence Types (ST) of *Xylella fastidiosa* from Costa Rica. ST53 and ST61, (each marked with an arrow) are shown relative to all other published sequence types of *X. fastidiosa*. CR *X. f.* subsp. *fastidiosa* defines STs from Costa Rica. Bootstrap values showing the separation of STs 53 and 61 from the other STs are shown (other bootstrap values are not relevant to the hypothesis being tested and are omitted for clarity).

**Figure 2:**
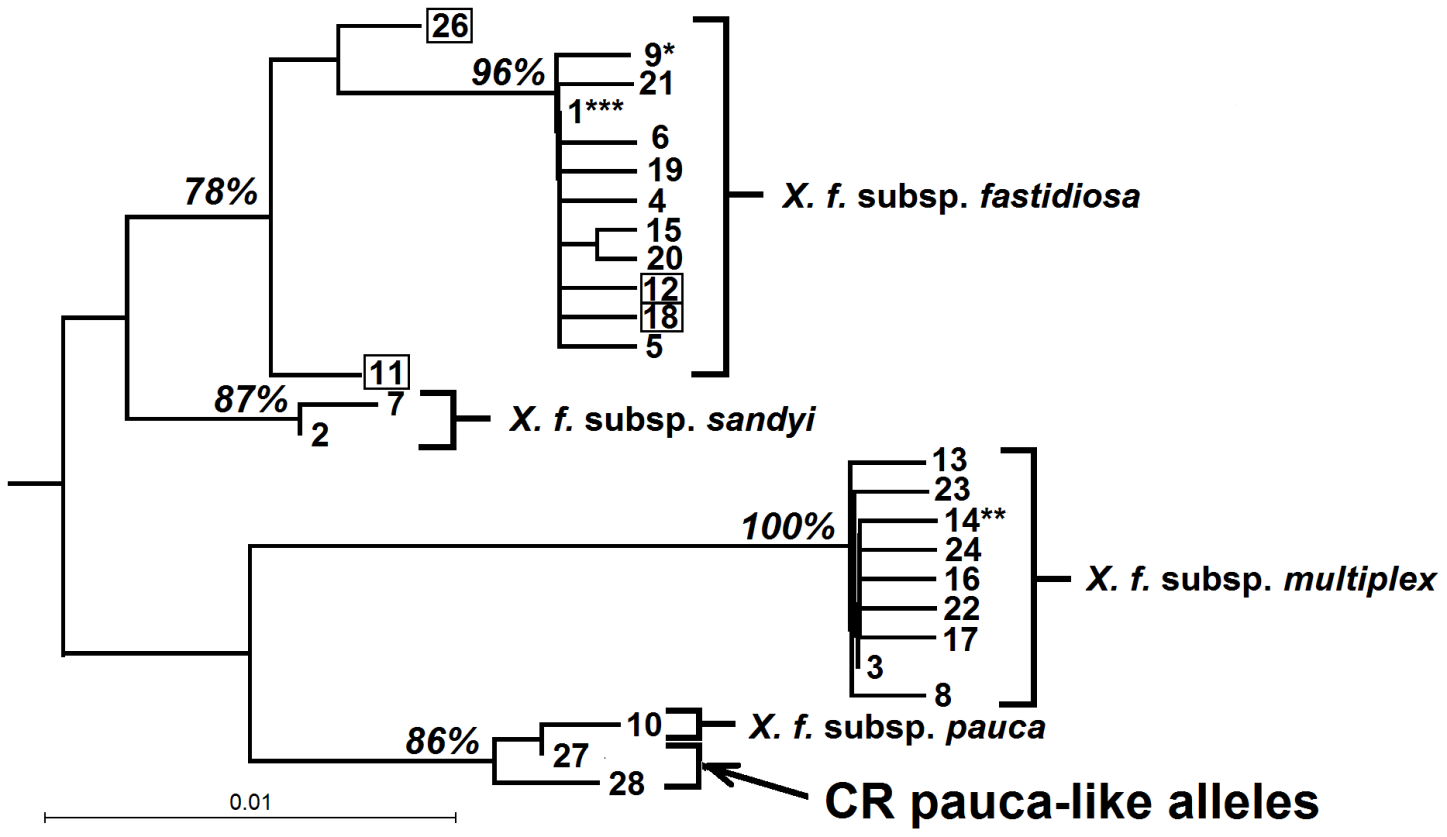
Distance tree of all known *Xylella fastidiosa pilU* alleles. Distance tree showing the close relationship of the novel pauca-like alleles 27 and 28 found in Costa Rica to the single allele (#10) found in the Brazilian *X. f.* subsp. *pauca*. The strong percentage bootstrap support for grouping these Costa Rican alleles with subsp. *pauca*, rather than with the other three subspecies is shown. The tree includes previously unpublished *pilU* data from Costa Rican *X. f.* subsp. *fastidiosa* isolates previously typed using MLST (11) (see text), including four new alleles (boxed). Alleles previously identified as involved in intersubspecific homologous recombination are indicated by: *, allele derived from *X. f.* subsp. *fastidiosa*, but found in recombinant *X. f.* subsp. *multiplex*; **, allele derived from *X. f.* subsp. *multiplex*, but found in *X. f.* subsp. *fastidiosa*; ***, allele characteristic of *X. f.* subsp. *fastidiosa*, but also found in recombinant *X. f.* subsp. *multiplex* and in *X. f.* subsp. *morus* [4], [12], [15]. Published *pilU* data from refs. [4], [13], [15].

The relationship of the two new STs to other sequenced isolates is shown in Figure 1, which includes all published MLST sequence types. Although PD0411 is identical to the other ST47 isolates at the MLST loci, its atypical *pilU* is apparent in Figure 2, which shows the relationship of all published *pilU* alleles, plus unpublished data from the 24 previously typed Costa Rican isolates [11]. These additional isolates carried five *pilU* alleles, one of which was identical to the common allele found in US *X. f.* subsp. *fastidiosa* (*pilU*#1), while the other four were new (Figure 2). The STs in which the four novel alleles they were recorded are: #11 in STs 20, 21 (one of two isolates), 33, 54, 56, 57 (plus ST61; see Table 1); #12 in STs 19, 21 (one of two isolates), 52; #18 in STs 17, 55; and #27, ST47.

### Evidence of *X. f. subsp. pauca* in Central America

**Figure 3:**
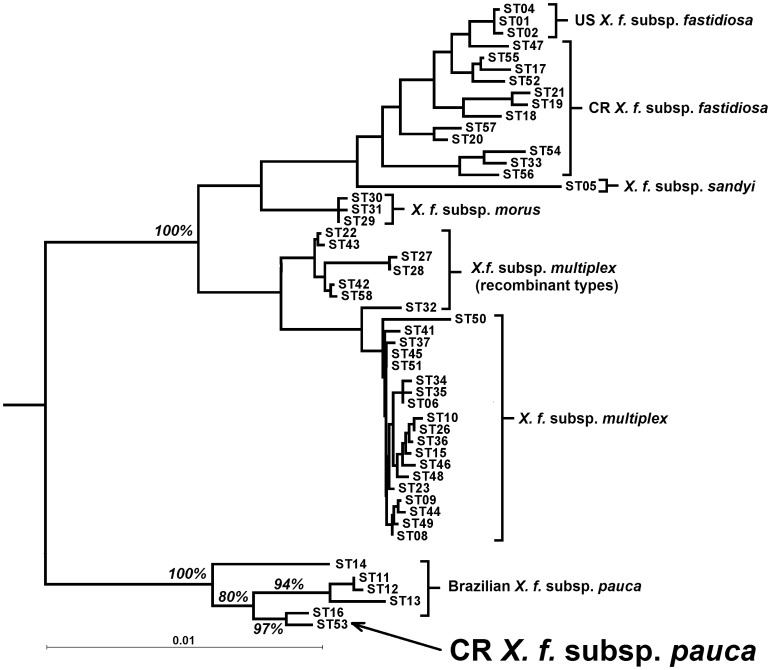
Distance tree of unique sequence types (STs) based on five MLST loci *leuA*, *petC*, *malF*, *holC*, and *gltT* plus the non-MLST locus *pilU*. Tree showing the grouping of the Costa Rica (CR) sequence type ST53 with the Brazilian *X. f.* subsp. *pauca* instead of grouping with the native (CR) *X. f.* subsp. *fastidiosa*. The 100% bootstrap support for this separation, and values within *X. f.* subsp. *pauca* are shown (other bootstrap values are omitted to aid clarity). The figure excludes STs that become identical to another ST when the alleles at *cysG* and *nuoL* are omitted.

ST53 was genetically distinct from all published STs from Costa Rica [11]: the alleles at all seven MLST loci differed substantially from those previously found in Costa Rica (ranging from 10–21 bp differences when compared to the most similar published *X. f.* subsp. *fastidiosa* alleles). In particular, the MLST genetic distance tree (Figure 1) shows that ST53 is genetically similar to *X. f.* subsp. *pauca*, and is significantly separated from the *X. f.* subsp. *fastidiosa* clade. Examining the sequences in more detail showed that five of the seven MLST alleles carried by ST53 were from *X. f.* subsp. *pauca* and not ancestrally from *X. f.* subsp. *fastidiosa*. Three of these alleles had previously been observed in *X. f.* subsp. *pauca* from Brazil (*leuA#*7, *petC*#6, *holC*#10) [10], and the other two were both 1 bp different from known *X. f.* subsp. *pauca* alleles (*malF*#15, 1 bp from #8; *gltT*#14, 1 bp from #8). In contrast, each of these five alleles was 10–17 bp different from the most similar *X. f.* subsp. *fastidiosa* allele. In addition, the non-MLST *pilU* allele (#27) was only 1 bp different from *X. f.* subsp. *pauca* allele #10 (Figure 2), but 10 bp different from the closest *X. f.* subsp. *fastidiosa* allele (*pilU*#11), the *pilU* allele found in most of the Costa Rica *X. f.* subsp. *fastidiosa* isolates. Thus there were only three differences across the 3549 bases of the six loci (0.1% divergence) between ST53 and the most similar *X. f.* subsp. *pauca* alleles from Brazil, compared to 79 differences across the 3549 bases (2.2% divergence) when compared to the equivalent set of the most similar *X. f.* subsp. *fastidiosa* alleles. These relationships are summarized in a tree based on these six loci (Figure 3), which clearly demonstrates that, based on these loci, ST53 is *X. f.* subsp. *pauca*. Comparing Figures 1 and 3, it can be seen that including the remaining two alleles (*cysG*#24 and *nuoL*#16) does not reverse the similarity to *X. f.* subsp. *pauca*, but it does reduce it, suggesting that these alleles have a somewhat different ancestry (see below).

Further evidence of the presence of *X. f.* subsp. *pauca* in Central America was provided by the isolate PD0411. The MLST signature (ST47) of this isolate from grapevine was identical to three other isolates from Costa Rica (from coffee and grapevine) and typical of *X. f.* subsp. *fastidiosa* [11]; however, at the non-MLST *pilU* locus, PD0411 carried an atypical allele (*pilU*#28; Table 1), whereas the other representatives of ST47 carried *pilU*#26, a new *X. f.* subsp. *fastidiosa pilU* allele found only in Costa Rica (Figure 2). While *pilU*#26 groups with the other *X. f.* subsp. *fastidiosa pilU* alleles, *pilU*#28 does not, instead grouping with the only known *X. f.* subsp. *pauca* allele, *pilU*#10. Specifically, *pilU*#28 differed from the *X. f.* subsp. *pauca* derived allele *pilU*#27 found in ST53 (described above) by only 2 bp, whereas it differed from *pilU*#26 (the most similar *X. f.* subsp. *fastidiosa* allele) by 9 bp. These results strongly support the hypothesis that PD0411, an *X. f.* subsp. *fastidiosa* isolate, gained its *pilU* allele via intersubspecific homologous recombination with *X. f.* subsp. *pauca*.

The tree of *pilU* alleles (Figure 2) also showed that of the five “typical” alleles found in the Costa Rican *X. f.* subsp. *fastidiosa* (#s 1, 11, 12, 18, 26), three of them (#s 1, 12, 18) grouped within a strongly supported clade of alleles (96% bootstrap support), while two of them (#s 11, 26) grouped outside of this clade (although still within a well-supported *X. f.* subsp. *fastidiosa* cluster). This genetic separation of alleles #11 and 26 from the other *X. f.* subsp. *fastidiosa* alleles was due to base differences at the 3′ end of the locus that are consistent with the introgression of sequence from *X. f.* subsp. *pauca* (or perhaps from the apparently related taxon described below). Testing for recombination using the modified introgression test [12] revealed significant evidence in the case of *pilU*#11 (p<0.01, based on 5′ vs. 3′ ratios of 0∶9 vs. 5∶2 for the differences between the target sequence and *pilU*#1 or #10, respectively). The effect was not significant for *pilU*#26 (p = 0.12 given 1∶10 vs 3∶2).

### Evidence of a previously unknown *X. fastidiosa* taxon

The introduction of *X. f.* subsp. *pauca* from South America into Central America explains most of the sequence data observed in the 10 atypical Costa Rica isolates, but not all. For example, it does not explain the origin of the *holC*#16 allele found in the ST61 (see Table 1). ST61 groups with *X. f.* subsp. *fastidiosa*, but is separated from the rest of the subspecies by a long branch (Figure 1), a pattern due to the presence of single atypical allele. This ST was identical at six of the seven MLST loci to another Costa Rica ST, ST56 [11], and both share the same *pilU*#11 allele. All of these shared alleles are typical of *X. f.* subsp. *fastidiosa*; however, ST61 differs from ST56 at *holC* (allele #16). The ST61 allele is 12 bp different from any previously observed *X. f.* subsp. *fastidiosa* allele (the most similar being *holC*#19), but it is also 11 bp from the closest *X. f.* subsp. *pauca* sequence (*holC*#11). Moreover, there is no indication that the allele is a recombinant mixture of these two subspecies. If the allele was such a recombinant mix then different portions of the allele would be similar to one or other of the subspecies. This pattern was not observed in *holC*#16. For example neither subspecies comes close to matching in the first 110 bp of the 379 bp locus (9 bp and 7 bp mismatches, respectively). Thus, based on the published subspecific variation, it is not clear where this allele originated.

A similar pattern is seen in both *cysG*#24 and *nuoL*#7 in ST53. The allele *cysG*#24 shows 15 bp and 21 bp differences from *X. f.* subsp. *pauca cysG*#11 and *X. f.* subsp. *fastidiosa cysG* #15, respectively, and both show particularly poor matches between positions 186–380 in the 600 bp locus (12 bp and 9 bp, respectively). A similar region lacking any apparent match to either subspecies is seen in the *nuoL*#16 allele of the same ST. This allele differs from *X. f.* subsp. *pauca nuoL*#7 and *X. f.* subsp. *fastidiosa nuoL* #1 by 17 bp and 20 bp, respectively, and neither match in the first 441 bp in a 557 bp locus (16 bp and 9 bp, respectively), although the *X. f.* subsp. *pauca* sequence does provide a good match for the remaining 115 bp (1 mismatch).

## Discussion

The analysis of 10 genetically atypical isolates of *X. fastidiosa* from Costa Rica showed that: (a) *X. f.* subsp. *pauca*, a subspecies considered native to South America, is present in Central America (as exemplified by ST53), and that it has introgressed into the native *X. f.* subsp. *fastidiosa* (as seen in PD0411); and (b) a second previously unknown form of *X. fastidiosa* has been identified from genetic material that has introgressed into *X. f.* subsp. *fastidiosa* (ST61) and *X. f.* subsp. *pauca* (ST53). This unknown form is closely related to *X. f.* subsp. *pauca*.

### *X. f. subsp. pauca* in Central America: native or introduced?

Based on prior analysis, it is clear that *X. f.* subsp. *fastidiosa* is native to Central America and was subsequently introduced into the US [11]. The data from the atypical Costa Rica isolates indicates a similar pattern of introduction of *X. f.* subsp. *pauca*, but this time into Central America from South America.

The alternative hypothesis is that *X. f.* subsp. *pauca* was not introduced, but that it is also native to Central America. Phylogenetic analysis suggests that the ancestors of *X. f.* subsp. *fastidiosa* and *X. f.* subsp. *pauca* diverged about 50,000 years ago [10], and evidence of genetic exchange between *X. f.* subsp. *fastidiosa* and *X. f.* subsp. *multiplex* in the US over the last 150 years [11]–[14] shows that when subspecies are in the same geographical area introgression occurs. This expectation is backed up by experimental data demonstrating high levels of transformation [22], [23]. If *X. f.* subsp. *fastidiosa* and *X. f.* subsp. *pauca* had been in contact for tens of thousands of years, it is probable that they would become thoroughly genetically mixed. However, even if somehow they were able to remain genetically distinct, it would be expected that the *X. f.* subsp. *pauca* in Central America would (a) become genetically distinct from those in Brazil, and (b) show genetic diversity. The sequence data suggest that neither of these expectations is supported. First, after detection of ST53 in oleander, additional infected plants were sampled to try to detect genetic variation. None was found (Table 1). Furthermore, although the introgressed *pilU* allele in PD0411 (*pilU*#28) differs by 2 bp from the *pilU*#27 in ST53, it is notable that these two base pairs are only 2 bp apart and that these specific nucleotides are uniquely shared with *pilU*#26 (carried by the other ST47 isolates). This pattern is a strong indicator of recombination, and so raises the possibility that *pilU*#27 recombined into ST47, but was then further modified by recombination with other *X. f.* subsp. *fastidiosa* creating *pilU*#28.

The second expectation is that if *X. f.* subsp. *pauca* had been present in Central America for any significant period of time, then it would become genetically differentiated from the Brazilian forms. This expectation is not supported: ST53 carries three alleles that are identical to alleles found in Brazil, and three others that differ by a single bp.

In summary, the data are consistent with the introduction of a single genotype of *X. f.* subsp. *pauca* from South America into Central America and are contrary to the pattern expected in a native subspecies. It also demonstrates the introgression of *X. f.* subsp. *pauca* sequence into the native *X. f.* subsp. *fastidiosa*, although to date this introgression appears to be limited.

### A possible new South American *X. fastidiosa* taxon

**Figure 4:**
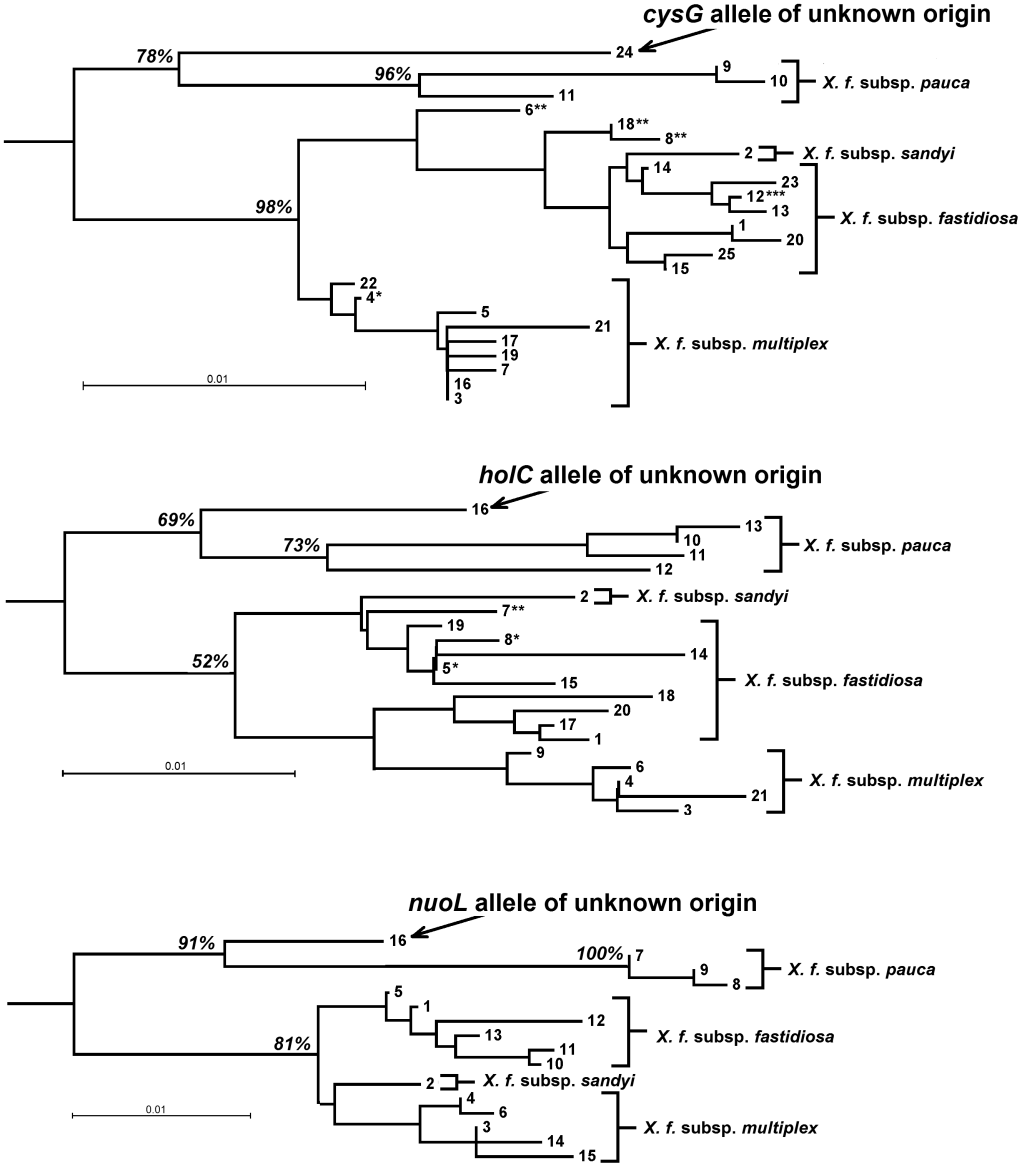
Distance trees of the alleles at the MLST loci *cysG*, *holC*, and *nuoL*. Tree showing that the “alleles of unknown origin” *cysG*#24, *nuoL*#16 and *holC*#16 map to a position close to, but distinct from, the South American subspecies *X. f.* subsp. *pauca*. The generally strong bootstrap support for grouping these alleles close to, but distinct from, subsp. *pauca*, rather than with the other three subspecies is also shown. In the *cysG* tree, *: allele #4 derived from *X. f.* subsp. *multiplex*, but found in *X. f.* subsp. *fastidiosa*; **: alleles #6, 8, 18 derived from *X. f.* subsp. *multiplex* and *X. f.* subsp. *fastidiosa* found in the recombinant *X. f.* subsp. *multiplex* and/or *X. f.* subsp. *morus*; ***: allele #12 derived from and found in *X. f.* subsp. *fastidiosa*, but also found in the recombinant *X. f.* subsp. *multiplex* [4], [12], [14], [15]. In the *holC* tree, *: alleles #5, eight derived from *X. f.* subsp. *fastidiosa*, but found in *X. f.* subsp. *morus*; **: allele #7 derived from *X. f.* subsp. *multiplex* and *X. f.* subsp. *fastidiosa* found in the recombinant *X. f.* subsp. *multiplex* [4], [12].

The origin of the three alleles *holC*#16, *cysG*#24, and *nuoL*#16 is unclear. A tree of each of the three loci, in each case rooted by the sequence from the *X. fastidiosa* isolated from pear in Taiwan [18], shows that they all group outside of the known subspecies, but closest to *X. f.* subsp. *pauca* (Figure 4). Despite this close relationship to *X. f.* subsp. *pauca*, the bootstrap support separating them from *X. f.* subsp. *pauca* is strong (*holC* 73%, *cysG* 96%, *nuoL* 100%), a pattern indicating that these novel allelic sequences originated from some unknown form of *X. fastidiosa* relatively closely related to *X. f.* subsp. *pauca*.

Our inability to identify the origin of *holC*#16, together with *cysG*#24, and *nuoL*#16, raises an important question concerning the extent to which we have a complete knowledge of the geographical diversity of *X. fastidiosa* within the Americas Based on the genetic distance relationships of these three alleles to all other known alleles (Figure 4), it appears that each comes from an unknown sister taxon to *X. f.* subsp. *pauca*. While this is speculative, the possibility highlights an important issue: our knowledge of the genetics of *X. fastidiosa* in South America is restricted to samples isolated in Brazil in areas growing citrus or coffee. There has been no genetic analysis of samples from native plants outside of the agricultural areas, nor have samples of *X. fastidiosa* been isolated from regions in the continent distant from Brazil. It is certainly possible that additional forms (perhaps even new subspecies) of *X. fastidiosa* may be found distributed across the continent.

### Invasion and host plant associations

The Brazilian *X. f.* subsp. *pauca* ST that is most similar to ST53 is ST16 (Figure 3). ST16 was isolated exclusively from coffee plants [10], One of the six Costa Rican isolates of ST53 was also isolated from coffee, while the other five were isolated from oleander, a species in the same order as coffee (Gentianales), but a different family (Apocynaceae vs. Rubiaceae). It is not known if ST16 can infect oleander, but it is possible that the introduction of some novel genetic material into ST53 (exemplified by the alleles cysG#24 and nuoL#16) has broadened the host range of *X. f*. subsp. *pauca*, which in Brazil appears to be largely restricted to coffee and citrus among agricultural and ornamental plants. The native hosts of *X. f*. subsp. *pauca* are unknown.

The introduction of *X. f.* subsp. *pauca* into Central America is of concern because of the ability of this subspecies to cause both coffee leaf scorch and citrus variegated chlorosis (CVC). *X. f.* subsp. *fastidiosa* also infects coffee, but the substantial drop in production due to coffee leaf scorch caused by *X. f.* subsp. *pauca* in Brazil [19], may be more serious than the losses due to the “crespera” disease typical of infection *by X. f.* subsp. *fastidiosa* [16], [20].

There has previously been a report of a CVC-like disease affecting citrus in Costa Rica [21], but we have no evidence that the *X. f.* subsp. *pauca* ST53 is capable of infecting citrus. The one citrus isolate that we have been able to type (CIT0208; see Table 1) was identified as ST61. Although this ST shows evidence of intersubspecific introgression at the *holC* locus (*holC*#16), the introgression is from the unknown taxon rather than *X. f.* subsp. *pauca*.

The possible existence of undiscovered genetic forms of *X. fastidiosa* in South America and the creation of new genetic forms through intersubspecific recombination is a concern because of the high probability that they will eventually be introduced into other regions through the movement of live plants, and that they may affect economically important crops either immediately or following introgression into the native form of *X. fastidiosa*. This appears to be the scenario unfolding in Costa Rica, and we can expect to see it repeated elsewhere.
